# Tracing pathways from antenatal to delivery care for women in Mumbai, India: cross-sectional study of maternity in low-income areas

**DOI:** 10.1016/j.inhe.2009.02.004

**Published:** 2009-09

**Authors:** Neena Shah More, Glyn Alcock, Ujwala Bapat, Sushmita Das, Wasundhara Joshi, David Osrin

**Affiliations:** a Society for Nutrition, Education and Health Action (SNEHA), Urban Health Centre, Chota Sion Hospital, 60 Feet Road, Shahunagar, Dharavi, Mumbai 400017, Maharashtra, India; b UCL Centre for International Health and Development, Institute of Child Health, 30 Guilford Street, London WC1N 1EH, UK

**Keywords:** Urban health, Private health care, Prenatal care, Maternity care, Slum, India

## Abstract

In many cities, healthcare is available through a complex mix of private and public providers. The line between the formal and informal sectors may be blurred and movement between them uncharted. We quantified the use of private and public providers of maternity care in low-income areas of Mumbai, India. We identified births among a population of about 300 000 in 48 vulnerable slum areas and interviewed women at 6 weeks after delivery. For 10,754 births in 2005–7, levels of antenatal care (93%) and institutional delivery (90%) were high. Antenatal care was split 50:50 between public and private providers, and institutional deliveries 60:40 in favour of the public sector. Women generally stayed within the sector and institution in which care began. Home births were common if women did not register in advance. The findings were at least superficially reassuring, and there was less movement than expected between sectors and health institutions. In the short term, we suggest an emphasis on birth preparedness for pregnant women and their families, and an effort to rationalize the process of referral between institutions. In the longer term, service improvement needs to acknowledge the private-public mix and work towards practicable regulation of quality in both sectors.

## 1 Introduction

We understand surprisingly little of the intricacies of healthcare in the developing nations of the South, particularly in the poorer parts of the cities.^1^ The need for more detailed information has been emphasised repeatedly over the last 20 years,^2^, ^3^, ^4^ with particular reference to the unexamined heterogeneity of low-income groups.^5^, ^6^, ^7^, ^8^, ^9^ In most cities, health services are available through a mosaic of private and public sector providers, and referrals are disorganized. The move towards private healthcare in India is unmistakeable.^10^, ^11^, ^12^ People appear to prefer it,^13^, ^14^, ^15^ particularly for outpatient care,^11^, ^16^ and it now accounts for over 80% of outpatient visits, 60% of inpatient expenditure and 40% of institutional births.^17^ The Indian private sector encompasses not-for-profit organizations (trusts, charities), the formal, ‘organised’ private sector (hospitals, practitioners, corporate diagnostic centres) and the informal, ‘unorganised’ sector.

Mumbai exemplifies the growing availability of healthcare. Its 16 million residents are presented with a bewildering choice of facilities,^18^ a choice that might even be considered excessive. It differs from many other cities, however, in that the Municipal Corporation of Greater Mumbai is a major healthcare provider. The corporation's department of public health administers six tertiary medical colleges, specialist hospitals and peripheral general hospitals, 26 maternity homes, 159 dispensaries and 176 health posts. Corporation hospitals contribute about 11 900 of Mumbai's estimated 40 000 hospital beds. At the same time, there has been an explosion in private healthcare,^1^, ^19^ with a wide range of provision from large, technologically replete hospitals, through small nursing homes with a few beds, to single-handed general practitioners. These practitioners are loosely regulated and have a range of qualifications and, presumably, skills and experience.^10^ In slum areas there is little distinction between the informal and formal sectors,^18^ and accessible private healthcare comes from nursing homes and practitioners seen as occupying the affordable end of the spectrum.

We are interested in maternity care for low-income women. Data on the number of clients who attend and move between public and private sector institutions are hard to find. Most opinions are based on experience at specific workplaces. For example, there is a general impression that movement up the primary healthcare chain is too common, such that higher grade hospitals in the public sector end up receiving clients registered in the private sector, and that they receive a substantial number of referrals or transfers from lower grade public sector facilities, such as maternity homes. Whether these flows are excessive is a matter for conjecture. It is certainly possible to find figures for individual institutions (we are collecting them in several public sector hospitals and maternity homes), but they are difficult to amalgamate and we do not have a clear, quantitative understanding of the care pathways that women follow during pregnancy and maternity. Fortunately, we now have access to information from a community-based maternity surveillance system covering a population of about 300 000 in vulnerable slum areas. Maternity experience is documented for all women who live in the sample areas, as part of the City Initiative for Maternal and Newborn Health.^20^

Our objectives for this analysis were to quantify the use of the public and private sectors for antenatal and delivery care, to quantify within this the use of specific types of institution and to quantify the movement of clients between them from antenatal care to delivery. We wanted to represent client flow in as visual a manner as possible, hoping that it would help us to locate points for intervention with maximal systemic and population benefits.

## 2 Methods

### 2.1 Participants

The study involved a sample of vulnerable areas in six municipal wards (F North, G North, H East, K West, M East, P North). We classified them as vulnerable in terms of having high proportions of social risk indicators (unemployment, groups in difficult circumstances, substandard housing), environmental indicators (open drainage, informal water supply, informal electricity supply) and health service utilisation indicators (infrequent interaction with community health volunteers, home deliveries). Of 92 possible areas, 48 were selected randomly in strata of eight per ward. Each area contained 1000–1500 households. A registration system was set up to identify births, stillbirths, neonatal deaths and maternal deaths.^20^

### 2.2 Procedures

Births were identified by 99 locally resident women, generally two per surveillance area, each covering an average of 600 households. Events were confirmed by 12 interviewers, responsible for four areas each, who visited mothers at home and arranged interviews at around 6 weeks after delivery. Interviews had a predominantly closed format, with questions on demography, socioeconomic factors and antenatal and delivery care. After an explanation of the data collection activities, participants were asked for verbal consent to be interviewed and assured of the confidentiality of data. Team members who encountered illness in mothers or infants had an ethical responsibility to recommend that they visit a health facility. Interviews were subject to a range of systematic and random checks for accuracy and completeness, both in the field and during entry into a relational database management system (Access; Microsoft Corp., Redmond, WA, USA). Information provided by participants remained confidential, and outputs did not include their names.

### 2.3 Statistical analysis

Data handling involved cross-tabulation and calculation of percentages. The flow map presentation used frequencies and was based on the legacy of Minard.^21^

## 3 Results

Table 1 summarises the sites of antenatal care for 10 754 births over 2 years, October 2005–September 2007. At least one antenatal care visit was made by 9983 (93%) women. Within Mumbai, antenatal care was split 50:50 between the public and private sectors. In the public sector, the commonest sites were municipal general hospitals (38% of public sector choices), followed by municipal maternity homes (31%) and tertiary hospitals (17%). Attendance for antenatal care at municipal health posts–the most community-focused institutions in the primary healthcare pyramid–was minimal. In the private sector, 58% of women chose a hospital and 42% a single-handed practitioner. Most women made the recommended three antenatal visits or more: 95% of women who had antenatal care in Mumbai and 89% of those who had it outside.

**Table 1:** Site of antenatal care for births in 48 slum areas of Mumbai, India, 2005–2007.

Location of care	*n*	(%)
In Mumbai	9145	(85)
Outside Mumbai	838	(8)
No antenatal care	771	(7)
Total	10754	(100)

Care in Mumbai		
Public sector	4541	(50)
Health post	67	(1)
Urban health centre	301	(3)
Maternity home	1412	(15)
General hospital	1713	(19)
Government hospital	266	(3)
Tertiary hospital	782	(9)
Private sector	4604	(50)
Private hospital	2669	(29)
Private practitioner	1935	(21)
Total	9145	(100)

Table 2 summarizes where Mumbai residents eventually gave birth. Of the 21% who chose to give birth outside the city (usually at their natal homes), 63% opted for institutional delivery. Within Mumbai there were 879 home births: 205 (23%) of these women had no antenatal care, 415 (47%) had antenatal care in the public sector and 259 (29%) in the private sector. Most women who ultimately delivered at home in Mumbai had actually registered for institutional delivery (675/879; 77%). Institutional delivery care was split 60:40 in favour of the public sector. General hospitals were again the commonest sites in the public sector (40%), but more births took place in municipal tertiary hospitals (26%) than in maternity homes (24%). Although 62 women said that they had delivered with a single-handed private practitioner, we have assumed that the deliveries took place at nursing homes.

**Table 2:** Site of delivery care for births in 48 slum areas of Mumbai, India, 2005–2007.

Location of delivery	*n*	(%)
Delivery in Mumbai		
Institutional	7663	(90)
Home birth	879	(10)
Total	8542	(100)

Delivery outside Mumbai		
Institutional	1383	(63)
Home birth	829	(37)
Total	2212	(100)

Institutional delivery in Mumbai		
Public sector	4685	(61)
Health post	0	
Urban health centre	211	(3)
Maternity home	1120	(15)
General hospital	1880	(24)
Government hospital	248	(3)
Tertiary hospital	1226	(16)
Private sector	2978	(39)
Private hospital	2978	(39)
Private practitioner	0	
Total	7663	(100)

**Figure 1:**
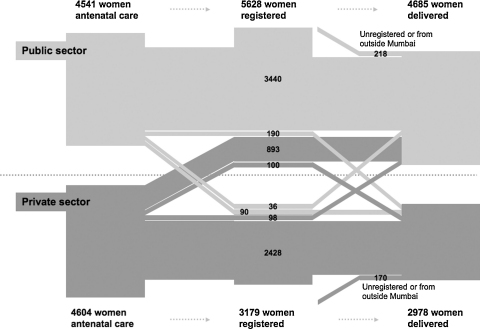
Traffic of clients between public and private sectors for antenatal care, registration and institutional delivery in Mumbai, India. Light grey: clients who began antenatal care in the public sector. Dark grey: clients who began in the private sector. Dotted line: notional divide between public and private sectors. The breadth of each path is proportional to the number of clients.

Figure 1 summarises the movement of clients between the public and private sectors. For the sake of clarity, it omits antenatal care and deliveries outside Mumbai. Reading from left to right, the flow map shows where women who had begun antenatal care in the public sector went on to register and subsequently deliver. The first visual impression is that clients generally stayed within the same sector. 7275 women had antenatal care, registered in advance for delivery and went on to deliver in an institution in Mumbai; 3440 (47%) did all three in the public sector and 2428 (33%) in the private sector. Second, the largest movement was from private sector antenatal care to public sector registration for delivery (993; 22% of those who had antenatal care in the private sector). Further breakdown of the data shows that 878 of these 993 women (88%) had chosen local care with a single-handed private practitioner. These practitioners tend not to provide delivery care, and clients would implicitly deliver elsewhere. Third, other movements between sectors did not substantially alter the picture. After registration, 290 clients moved from the public to the private sector and 134 from private to public, a net shift of 156 from public to private, representing 3% of public sector and 5% of private sector deliveries. Likewise, the numbers of clients who arrived for institutional delivery without either antenatal care or having registered in advance were limited: 5% (218/4685) of public sector and 6% (170/2978) of private sector deliveries.

**Figure 2:**
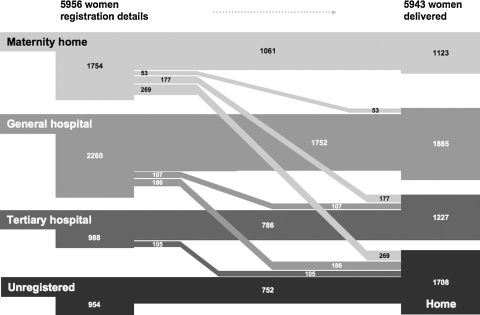
Progress of clients between types of public sector institution from registration to delivery in Mumbai, India. The breadth of each path is proportional to the number of clients. Urban health centre clients are omitted due to small numbers. Flows in which fewer than 25 women were involved are not included: unregistered to institutional delivery, tertiary to general hospital, tertiary hospital to maternity home, general hospital to maternity home.

Moving from a sectoral to an institutional level, the least movement was seen between private hospitals, in which 3011 (28%) deliveries took place. Of the 3043 women who registered at a private hospital, 2503 (82%) delivered at the same hospital. In the public sector too, most women delivered at the institution at which they had registered to do so. Concordance between registration and delivery was high in municipal tertiary hospitals (786/988; 80%) and general hospitals (1752/2260; 78%), but lower at municipal maternity homes (1061/1754; 60%) and urban health centres (189/325; 58%). Figure 2 summarises the progress of clients from registration to delivery in major public sector institutions and includes women who, although they lived in the study areas, had antenatal care, registration or delivery outside Mumbai. Most women who did not register at institutions went on to give birth at home (752/954; 79%). Discordance between registration and delivery was most marked for maternity homes and tertiary hospitals. The major traffic was from maternity home or tertiary hospital registration to home delivery (15% and 11%, respectively) and from maternity home or urban health centre registration to delivery at a tertiary hospital (10% and 22%, respectively).

The reasons for some of these flows are presented in Table 3. The most common reasons for home delivery, after planning a delivery at a municipal maternity home or tertiary hospital, were that the progress of labour was too rapid for the woman to reach the institution in time or that there was nobody available to go with her. The reputation of public sector staff for poor communication skills was also a disincentive. The explanation that home birth was customary suggests that registration may be a fairly automatic part of antenatal care that is not necessarily taken up.

**Table 3:** Reasons for discordance between site of registration and site of delivery.

Reason	*n*	(%)
Home birth after registration at a maternity home or tertiary hospital		
Not enough time to reach hospital	66	(21)
Nobody to accompany	60	(19)
Afraid of hospital staff	22	(7)
Home birth customary	19	(6)
Nobody to look after children or home	18	(6)
Told to return at a later stage of labour	14	(4)
Household work responsibilities	11	(3)
Transport not available	9	(3)
No problems, so did not see need	8	(3)
Too weak to reach hospital	7	(2)
Cost	6	(2)
Did not have necessary papers	4	(1)
Hospital too far away	3	(1)
Hospital closed for renovation or strike	3	(1)
Family did not permit hospital delivery	3	(1)
Other	23	(7)
Unspecified	44	(14)
Total	320	(100)

Reason for delivery at a tertiary hospital after registration at a maternity home or urban health centre		
Tertiary hospital near home	134	(54)
Medical indication	42	(17)
Refused admission: missing reports, no bed	17	(7)
Went to maternity home in another part of Mumbai	16	(6)
Lack of equipment or bed: operative, transfusion, neonatal care, electricity	4	(2)
Poor opinion of registration facility: care, attitude of staff, hygiene	3	(1)
Transferred for unclear reason	3	(1)
Told to return at a later stage of labour	3	(1)
Institution closed for renovation or strike; doctor unavailable	3	(1)
Family advice or decision	2	(1)
Not enough time to reach institution	1	(<1)
Two-child norm (charge for a birth order higher than two)	1	(<1)
Unspecified	18	(7)
Total	247	(100)

The most common reason for delivery at a municipal tertiary hospital after registration at a maternity home or urban health centre was that the woman lived not far from Lokmanya Tilak Municipal General Hospital at Sion and had decided to go there (54%). The second most common reason was that there was a medical indication for referral or transfer. We are unable to judge the appropriateness of this. However, we did classify women as having encountered a serious medical or obstetric problem on the basis of the description of conditions that clinical best practice would consider as unequivocally requiring emergency clinical care. These included jaundice, malpresentation, twin pregnancy, antepartum haemorrhage, prolonged labour, failure to progress, obstructed labour, convulsion, high blood pressure, postpartum haemorrhage, retained placenta, exhaustion or unconsciousness, or requirement for blood transfusion. Ten of the 42 women who reported a move to a tertiary hospital for medical reasons fell into this category. When a woman moved from a maternity home or urban health centre to a tertiary hospital, she might have been sent before or after actual admission. Referral before admission, sometimes from the gate of the institution, was made in 57% of cases (140/247). Conversely, 57% of transfers for medical indications were made after a period of admission (24/42).

## 4 Discussion

In our study of over 10 000 births to women living in vulnerable slum areas of Mumbai, the findings were at least superficially reassuring. Most women used health facilities for antenatal care, registration for delivery and delivery itself. Once they had entered the healthcare system antenatally, they were likely to remain in it until delivery. Both public and private sectors were used and, with the exception of a switch from single-handed private practices to public facilities for registration and delivery, there was less movement between sectors or health institutions than expected.

The strengths of the study were that it was community-based and could document sources of care for all women who gave birth, and that it involved poor families living in vulnerable slum areas. Weaknesses included the possibility of missed births (few, in our estimation) and the need to rely on client descriptions of indications for referral, which would reflect perceptions of service quality rather than clinical guidelines. The study did not involve wealthier residents of Mumbai; use of the public sector dwindles with rising wealth, and it is now the de facto provider for poorer people.

How should the observed patterns of care be interpreted? The big picture suggests that something is going right (see Figure 1). Women received antenatal and delivery care in a system that weaves together multiple levels and sources of service. This is true across urban India, where 91% of women now have antenatal care and 68% institutional delivery.^22^ We propose that the actual pattern can be explained by three narratives: a perceived need for maternity care, a preference for private healthcare (but a ceiling on ability to pay) and a view of public sector care in which at least two positions compete.

Urban residents now perceive a need for medical care during pregnancy and birth. We think that this perception combines an understanding of health issues and an identification of urban life with the project of modernity. This extends to those who deliver outside Mumbai; the tradition of return to the natal home for confinement may disrupt continuity of care,^13^ but almost two-thirds of women who left the city still delivered at a health facility. The preference for private healthcare is clear.^11^, ^16^, ^23^ We have demonstrated a steady increase in private sector maternity care with rising socioeconomic status in the same group of mothers involved in this study; the trend was seen even among poor families, who would be viewed from outside as a relatively homogeneous slum population.^24^ Private care offers proximity, affability, shorter queues and more communication. Practitioners set their prices to suit their clientele and many of their clinics are in urban slums, so that the cost of antenatal care is low.^11^

At a certain point on a sliding scale of spending power, however, private care becomes too costly. We think that perceptions of public sector maternity care are confused; people may not mean exactly what they say, or know exactly what they think. The ubiquitous denigration of Municipal Corporation institutions that fills newspapers and characterises focus groups (N. Shah More, 2008, unpublished) is not supported by usage data and individual interviews. It seems that the primary disincentives to usage are the expectation that care will be fraught with delays, directives and dismissiveness. Although these are precisely the features driving the move to the private sector, they are not necessarily issues of clinical quality. Incidental support for this argument is that women who had private sector antenatal care tended to move to the public sector for delivery. They did not dismiss it altogether, they used it. As long as provision of care is reasonable, a suboptimal experience of care is tolerated.^18^, ^25^ We have evidence of this attitude from exit interviews with maternity care clients, who repeatedly emphasised that public sector services were good ‘for poor people’ (S. Shende, 2008, unpublished).

Although the general picture looks coherent, there are potential quality gaps. Home births were not uncommon, and although most were to women who had not registered at a health facility, a substantial number had received antenatal care, mostly in the public sector. This was particularly true of women who had registered in a municipal maternity home or tertiary hospital, but ultimately gave birth at home. Many of these women said that they had not had time to reach the hospital, or had nobody to accompany them, and clearly this is a problem that might improve with some advance planning and a shorter reaction time on the part of family members.^23^, ^26^

There was also a tendency to register at a maternity home or urban health centre, but deliver at a tertiary hospital. The most obvious explanation for this, and the one we feel is likely, is that registration for delivery is part of the package of antenatal care. In many cases, women had chosen antenatal care at a maternity home near to their home, had put their name down for delivery, but had then chosen to go to a nearby tertiary hospital for delivery. Our sense is that many of them had never intended to follow the registration. Antenatal care at tertiary hospitals involves long waits at busy facilities, while maternity homes tend to be less crowded. The benefits of proximity, friendliness and relative calm tip the balance in favour of local maternity homes and urban health centres for antenatal care, but the perceived benefit of technical quality tips it towards tertiary hospitals for delivery.

Where should attention be focused to improve quality of care and outcomes?^27^ Perhaps a complex system generates an appearance of rationality, and it is not the outcomes we should look at but the process. We are conducting more detailed studies of women's experience of care (in contradistinction to the provision of care),^28^ the dynamics of choice of private provider, care-seeking when problems arise, home births and the use of traditional birth attendants in contemporary urban life, and the process of referral between institutions. In the short term, we suggest an emphasis on two things. First, advice on birth preparedness for pregnant women and their families, so that they know where they are going for delivery, how long it will take to get there and what means of transport they will use, so that other family members can be ready to react when labour begins. Secondly, consideration of ways to rationalise, protocolise and systematise referral and transfer so that health institutions are aware of the big picture and their contribution to it.

In the longer term, we need to view public sector service improvement as part of a picture that includes the private sector,^29^ and to adopt a rational approach to accreditation and collaboration between them, with practicable regulation of quality in both sectors. This is overdue, but not easy. Given the attrition to the private sector, the municipal health system may come to serve an increasingly poor segment of society, as well as a higher risk group of clients. This is likely to be de-motivating for public sector health workers and will contribute to maintaining the narrative of criticism that informs care-seeking.

## Funding

The City Initiative for Newborn Health is supported by the ICICI Centre for Child Health and Nutrition. This work was also supported by The Wellcome Trust (Grant number 081052).

## Role of the funding source

The sponsors had no role in the study design, data collection, analysis, interpretation or writing of the article. DO had full access to all study data and took final responsibility for the decision to submit for publication.

## Conflicts of interest

None declared.

## Ethical approval

Data for the study originated from a trial approved by the Municipal Corporation of Greater Mumbai, the Independent Ethics Committee for Research on Human Subjects (Mumbai committee, reference IEC/06/31) and the ethics committee of the Institute of Child Health and Great Ormond Street Hospital for Children, London, UK.

## Authors’ contributions

NSM was the project coordinator, contributed to the design of the study and participated in data analysis and interpretation; GA worked on the analysis and interpretation of the data and co-wrote the first draft of the article; UB and SD contributed to the study design, were responsible for the management of data collection and entry, and participated in data analysis and interpretation. WJ is the director of SNEHA and had overall responsibility for Mumbai work as well as contributing to the interpretation of the data; DO contributed to the study design, data management, analysis and interpretation, and co-wrote the first draft of the article. All authors contributed to critique and modification of the manuscript and read and approved the final version. DO is guarantor of the paper.
